# Respiratory Viral Co‐Infections in SARS‐CoV‐2 Positive Patients in Burkina Faso: A Cross‐Sectional Study

**DOI:** 10.1002/hsr2.72339

**Published:** 2026-04-16

**Authors:** Benjamin W. O. Kaboré, Nina Gouba, Abdoul Kader Ilboudo, Assana Cissé, Moussa Lingani, Cathérine Sawadogo, Ezéchiel Wendinda Ouédraogo, Jean Bienvenue Ouoba, Madi Savadogo, Zékiba Tarnagda

**Affiliations:** ^1^ Institut de Recherche en Sciences de la Santé‐Laboratoire National de Référence Grippe Ouagadougou Kadiogo Burkina Faso; ^2^ Département de Biochimie‐Microbiologie, UFR‐SVT Université Nazi Boni Bobo‐Dioulasso Burkina Faso; ^3^ Institut de Recherche en Sciences de la Santé‐Direction Régionale du Centre‐Ouest Nanoro Nando Burkina Faso; ^4^ Centre Universitaire polytechnique de Manga Nazinon Burkina Faso; ^5^ Direction Générale des Services Vétérinaires, Ministère de l'Agriculture, des Ressources Animales et Halieutiques Ouagadougou Kadiogo Burkina Faso; ^6^ Fundamental and Applied Research for Animals and Health, Faculty of Veterinary Medicine University of Liege Liege Belgium

**Keywords:** Burkina Faso, respiratory viruses, SARS‐CoV‐2, viral co‐infection

## Abstract

**Background:**

The burden of viral co‐infections among COVID‐19 patients is underexplored in low‐resource settings such as Burkina Faso. This study aimed to determine the prevalence and associated factors of respiratory viral co‐infections among laboratory‐confirmed COVID‐19 patients.

**Methods:**

A cross‐sectional study was conducted from January 1 to December 31, 2023, across 13 sentinel sites in Burkina Faso. Patients presenting with influenza‐like illness or severe acute respiratory infection had nasopharyngeal and/or oropharyngeal swabs collected and tested for SARS‐CoV‐2 and other respiratory viruses using multiplex real‐time RT‐PCR. Descriptive statistics, univariate, and multivariate logistic regression analyses were performed to identify predictors of viral co‐infections.

**Results:**

Among 201 SARS‐CoV‐2‐positive patients enrolled (median age: 1 year; interquartile range: 0.5–7). Co‐infections was identified in 48.8% (95% CI: 41.8–55.6) of cases and a single co‐infections was detected in 33.8% (95% CI: 27.5–40.6) of patients. Human rhinovirus (17.9%), adenovirus (14.4%), respiratory syncytial virus (11.4%), and influenza A/B (9.9%) were most frequently detected. In multivariate analysis, children aged 6 months to 14 years had higher odds of co‐infection (aOR 2.7; 95% CI: 1.2–5.9; *p* = 0.013). Fever (aOR 2.6; 95% CI: 1.3–5.5; *p* = 0.008) and hospitalization (aOR 4.3; 95% CI: 1.6–12.0; *p* = 0.005) were also independently associated with co‐infection.

**Conclusion:**

Viral co‐infections were frequent among SARS‐CoV‐2 positive patients, particularly in children and hospitalized individuals. Strengthening multiplex diagnostic capacity and integrated respiratory virus surveillance in resource‐limited settings may improve clinical management and inform public health response strategies.

## 1 Introduction

The COVID‐19 pandemic has significantly influenced the epidemiology of respiratory infections worldwide [1]. While SARS‐CoV‐2 has been extensively studied in isolation, its interactions with other respiratory viruses, including the potential for co‐infections, remain poorly understood, particularly in low‐resource settings such as Burkina Faso.

Co‐infections with multiple respiratory viruses can complicate diagnosis, potentially worsen clinical outcomes, and pose challenges for infection control [2, 3]. Previous studies have shown that co‐infections may either exacerbate or mitigate disease severity, depending on the viral agents involved [4, 5]. The true burden of viral co‐infections in sub‐Saharan Africa remains underreported due to limited diagnostic capacity and surveillance infrastructure.

In Burkina Faso, the national influenza sentinel surveillance system has provided a valuable platform for monitoring respiratory virus circulation since its establishment. With the integration of SARS‐CoV‐2 testing into this system, it is now possible to investigate patterns of co‐infection involving this novel virus. Circulation of a wide range of respiratory viruses, including influenza viruses, respiratory syncytial virus (RSV), human parainfluenza viruses (HPIV), human rhinoviruses (HRV), and adenoviruses (AdV), has been documented across various age groups, particularly among children [6, 7].

To our knowledge, this is the first nationwide sentinel surveillance analysis in Burkina Faso examining co‐infections between SARS‐CoV‐2 and other respiratory viruses in the post‐pandemic period. This study aimed to determine the prevalence and types of respiratory viral co‐infections among SARS‐CoV‐2‐positive patients and to identify demographic and seasonal factors associated with co‐infection in the context of routine surveillance in Burkina Faso in 2023.

## 2 Methods

### 2.1 Ethics Statement

The study was conducted in accordance with the Declaration of Helsinki [8] and approved by the Comité d'Éthique pour la Recherche en Santé (CERS) of Burkina Faso (Approval No. 2025‐10‐473). Oral informed consent was obtained from all adult participants and from parents or legal guardians for all minors prior to enrollment.

### 2.2 Study Design and Setting

We conducted a cross‐sectional study using data from the national influenza sentinel surveillance system in Burkina Faso, covering the period from January 1 to December 31, 2023. The study included all SARS‐CoV‐2‐positive patients identified during this period (*n* = 201). No formal sample size calculation was performed due to the observational nature of the study. This study was conducted according to the STROBE guidelines for cross‐sectional studies (Supporting Information file 1) [9].

**Figure 1:**
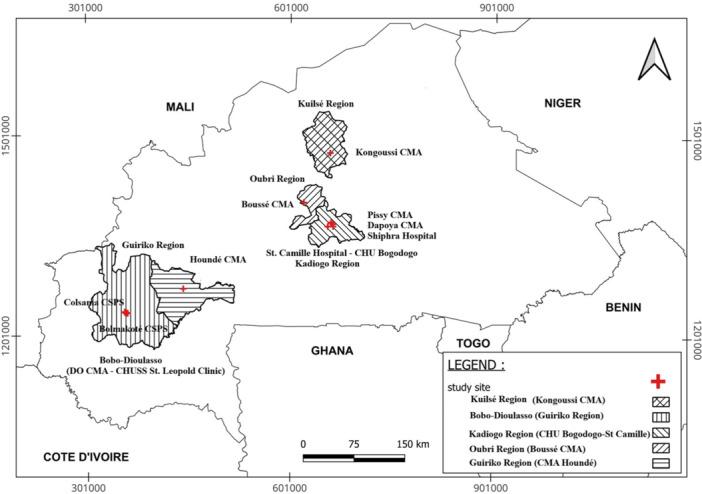
Map of acute respiratory infection surveillance sites in Burkina Faso, 2023. The sentinel sites are located at: Boussé CMA (Oubri region), Kongoussi CMA (Kuilsé region), CHU Bogodogo and St. Camille Hospital (Kadiogo region), as well as in Bobo‐Dioulasso (Bolomakoté CSPS and Colsama CSPS) and Houndé CMA (Guiriko region). The six pilot sites are located at: Ouagadougou (Pissy CMA, Dapoya CMA, and Schiphra Hospital, Kadiogo region) and Bobo‐Dioulasso (St. Leopold Clinic, DO CMA, and CHU Sanou Sourou, Guiriko region).

The study was carried out within the national influenza surveillance network, which comprised 13 health facilities across four regions (Kadiogo, Oubri, Kuilsé, and Guiriko) (Figure 1). Seven were established sentinel sites, including two primary health‐care facilities dedicated to influenza‐like illness (ILI) surveillance and five hospitals responsible for severe acute respiratory infection (SARI) surveillance. In 2023, six additional pilot sites were integrated through the AFROSCREEN project to strengthen SARS‐CoV‐2 genomic surveillance. Overall, the network included three teaching hospitals, four district hospitals, and six primary health‐care facilities. SARI cases were recruited at teaching and district hospitals, whereas ILI cases were enrolled at primary health‐care facilities. Surveillance activities were coordinated by the Ministry of Health in collaboration with the National Influenza Reference Laboratory (NIRL) at the Institute of Research in Health Sciences (IRSS).

### 2.3 Case Definitions and Eligibility

Patients presenting with respiratory symptoms were assessed using standardized WHO case definitions [10].

- **ILI**: acute respiratory infection with fever ≥ 38°C and cough, onset within the previous 10 days, in an outpatient setting.
- **SARI**: acute respiratory infection with fever ≥ 38°C and cough, onset within the previous 10 days requiring hospitalization.

Inclusion criteria were patients meeting either ILI or SARI definitions. Only community‐acquired cases were included. Exclusion criteria were: refusal of consent or specimen collection, severe unrelated chronic illness (e.g., heart failure, chronic respiratory failure, active malignancy, and end‐stage renal disease) or missing SARS‐CoV‐2 test results.

Comorbidities including cardiovascular diseases, malnutrition, asthma, diabetes, neurological disorders, and other chronic conditions were recorded at admission.

Severe outcomes were defined as respiratory distress, dyspnea, lethargy, convulsions, oxygen therapy, ICU admission, and/or in‐hospital death. Oxygen therapy included nasal cannula, face mask, non‐invasive, or invasive mechanical ventilation.

Abnormal chest X‐ray findings included alveolar consolidation, interstitial infiltrates, ground‐glass opacities, pleural effusion, or other pathological findings reported by radiologists.

The dry season covers the period from October to April, and the rainy season from May to September.

### 2.4 Data and Specimen Collection

All sites followed a standardized protocol for patient recruitment and sample collection. Personnel received training on case definitions and sample handling. Demographic and clinical data were recorded using standardized case report forms (Supporting Information file 2). Nasopharyngeal and/or oropharyngeal swabs were collected using Copan Universal Transport Medium (UTM) kits (Copan Diagnostics, Italy).

### 2.5 Sample Transport

Specimens were transported at 4°C and delivered within 48 h to the NIRL via the integrated biological specimen referral system (SITEB) using national courier services (La Poste Burkina Faso) [11]. On arrival, samples were logged, assessed for quality, and aliquoted into three portions: one for immediate testing, and two stored at −80°C for future analyses.

### 2.6 Laboratory Analysis

#### 2.6.1 SARS‐CoV‐2 and Influenza Virus Detection

Total nucleic acids were extracted using the 3DMed ANDiS 350 automated system (3D Biomedicine Science & Technology Co. Ltd., China). Initial screening for SARS‐CoV‐2 and Influenza A/B was performed using CDC‐validated real‐time retro‐transcription polymerase chain reaction (RT‐PCR) assays [12] on the Quant Studio 5 equipment (Thermo Fisher Scientific, USA). Influenza A‐positive samples were subtyped for A (H1N1) pdm09 and A (H3N2), and influenza B samples were characterized for the B/Victoria lineage.

#### 2.6.2 Multiplex Detection of Respiratory Viruses

Samples were also tested using the FTD Respiratory Pathogens 21 multiplex RT‐PCR assay (Siemens Healthineers, Germany). The assay detects influenza A/B, human rhinovirus (HRV), adenovirus (HAdV), parainfluenza viruses 1‐4 (HPIV 1‐4), human metapneumovirus (HMPV‐A/B), respiratory syncytial virus (RSV‐A/B), bocavirus (BoV), parechovirus (HPeV), enterovirus (EV), and seasonal human coronaviruses (HCoV 229E, NL63, HKU1, OC43). Detection of viral RNA was interpreted as co‐infection regardless of cycle threshold (Ct) value, as viral load data were not incorporated into the analytical model.

#### 2.6.3 Quality Assurance and Data Integration

All PCR assays included internal amplification and extraction controls. Routine quality assurance procedures ensured accuracy and reproducibility. Results were validated by trained NIRL personnel and integrated into the Ministry of Health's electronic surveillance system (STELaB).

### 2.7 Statistical Analysis

Data were entered in Microsoft Excel and analyzed using Stata version 18.0 (StataCorp, USA). Categorical variables were summarized as frequencies and percentages. Associations were assessed using the Chi‐square or Fisher's exact tests, as appropriate.

Univariate logistic regression evaluated factors associated with viral co‐infection; crude odds ratios (ORs) with 95% confidence intervals (CIs) were calculated. Variables with *p* < 0.20 in univariate analyses or considered clinically relevant were included in multivariable logistic regression. All adjusted odds ratios (aORs) are reported with 95% CIs. Backward stepwise selection based on the Akaike Information Criterion (AIC) was applied, adjusting for age, sex, region, and clinical variables. Multicollinearity was assessed using variance inflation factors (VIF). Model calibration and discrimination were evaluated using the Hosmer‐Lemeshow test and area under the ROC curve (AUC), respectively. Complete‐case analysis was performed; missing data were < 5%. Statistical analyses were performed in accordance with the Guidelines for reporting statistics in clinical research in urology [13]. Statistical significance was based on *p*‐value < 0.05.

## 3 Results

A total of 201 patients testing positive for SARS‐CoV‐2 were included in the study. Of these, 76.6% (*n* = 154) presented with ILI, while 23.4% (*n* = 47) were classified as having SARI. The study population was predominantly pediatric, with a median age of 1 year (interquartile range: 0.5–7). Children aged 6 months to 14 years were the most represented group, accounting for 53.2% (107/201) of the cohort, and 57.7% (*n* = 116) were female. The most common clinical symptoms were cough (96.5%) and fever ≥ 38°C (69.2%), while SARI cases were more likely to present with respiratory distress (44.7%) and malnutrition (28.9%). Regarding management, 30.8% of patients received antibiotic therapy during hospitalization, with ceftriaxone being the most frequently prescribed antibiotic, administered in 93.6% of patients receiving antibiotics. All demographic and clinical characteristics are summarized in Table 1.

**Table 1:** Demographic and clinical characteristics of 201 SARS‐CoV‐2‐positive cases.

Variables	ILI (*n*, %) 154 (76.6)	SARI (*n*, %) 47 (23.4)	Total positive SARS‐CoV‐2 *n* = 201
Age group			
Median, year (IQR)	1 (0.5–18)	1 (0.4–3)	1 (0.5–7)
0–6 months	32 (20.8)	13 (27.7)	45 (22.4)
6 months–14 years	82 (53.2)	25 (53.2)	107 (53.2)
≥ 15 years	40 (26.0)	9 (19.1)	49 (24.4)
Sex			
Male	70 (44.9)	15 (33.3)	85 (42.3)
Female	86 (55.8)	30 (63.8)	116 (57.7)
Area of residence			
Rural	1 (0.6)	12 (26.7)	13 (6.5)
Urban	155 (99.4)	33 (73.3)	188 (93.5)
Region			
Guiriko	110 (70.5)	26 (57.8)	136 (67.6)
Kadiogo	46 (29.5)	14 (31.1)	60 (29.8)
Oubri	0 (0)	5 (11.1)	5 (2.4)
Vaccination anti‐COVID‐19	7 (4.5)	1 (2.1)	8 (3.9)
Comorbidities			
Asthma	1 (0.6)	0 (0)	1 (0.5)
Hypertension	5 (3.2)	0 (0)	5 (2.5)
Diabetes	2 (1.3)	0 (0)	2 (1.0)
Obesity	2 (1.3)	0 (0)	2 (1.0)
Sickle cell disease	1 (0.6)	0 (0)	1 (0.5)
Malnutrition	0 (0.0)	13 (28.9)	13 (6.7)
Prematurity	0 (0.0)	1 (2.2)	1 (2.2)
HIV	0 (0.0)	1 (2.2)	1 (2.2)
Onset‐symptoms			
Fever (≥ 38°C)	119 (77.3)	20 (42.6)	139 (69.2)
Lethargy	0 (0.0)	3 (6.7)	3 (1.5)
Cough	155 (99.4)	42 (89.4)	194 (96.5)
Dyspnea	1 (0.6)	2 (4.4)	3 (1.5)
Respiratory distress	3 (1.9)	21 (44.7)	24 (12)
Sore throat	8 (5.2)	0 (0.0)	8 (4.0)
Loss of taste	4 (2.6)	0 (0.0)	4 (2.0)
Loss of smell	3 (1.9)	0 (0.0)	3 (1.5)
Convulsion	0 (0.0)	4 (8.9)	4 (2.0)
Diarrhea	5 (3.2)	12 (26.7)	17 (8.5)
Vomiting	4 (2.6)	11 (24.4)	15 (7.5)
Antibiotic before hospitalization		49 (24.3)	49 (24.3)
Antibiotic during hospitalization		62 (30.8)	62 (30.8)
Antibiotic administered			
Ceftriaxone	—	44 (93.6)	44 (21.9)
Gentamycin	—	9 (4.5)	9 (4.5)
Ampicillin	—	1 (2.1)	1 (2.1)
Nystatin	—	2 (4.2)	2 (4.2)
Amoxicillin	—	6 (12.7)	6 (12.7)
Abnormal X‐ray	—	8 (4.0)	8 (4.0)
Oxygen therapy	—	11 (5.5)	11 (5.5)
ICU admission	—	12 (6.0)	12 (6.0)
Severe outcome			
Non‐severe	150 (96.8)	17 (37.8)	167 (83.5)
Severe	0 (0.0)	33 (16.5)	33 (16.5)
Season^a^			
Dry season	112 (71.8)	32 (68.1)	144 (71.6)
Rainy season	44 (28.2)	13 (28.9)	57 (28.3)

Abbreviations: ICU = Intensive care unit, ILI = Influenza‐like illness, SARI = severe acute respiratory infection, SD = standard deviation.

^a^ Dry season: October‐April; Rainy season: May‐September.

Overall prevalence of viral co‐infections was 48.8% and 33.8% of patients had a single additional pathogen detected, while 17.4% exhibited multiple co‐infections (ranging from two to four additional viruses) (Table 2). Rhinovirus was the most frequently detected co‐pathogen (17.9%; 95% CI: 13.1–23.8), followed by Adenovirus (14.4%; 95% CI: 10.2–20.0) and RSV (11.4%; 95% CI: 7.7–16.6). Influenza viruses (A and B) were present in 10.0% (95% CI: 6.4–14.9) of patients, with a dominance of the A (H1N1) pdm09 subtype (7.0%; 95% CI: 5.5–13.5). Seasonal human coronaviruses (HCoV) and HPIV were identified in 7.0% (95% CI: 4.1–11.7) and 4.5% (95% CI: 1.6–7.3) of cases, respectively.

**Table 2:** Repartition of viral co‐infection in SARS‐CoV‐2 patients.

Co‐infection	Total detection (*n*, %)	(95%, CI)
Influenza A and B	20 (10.0)	6.4–14.9
Influenza A	18 (8.9)	5.5–13.5
Influenza A(H1N1)pdm09	14 (6.9)	3.4–10.5
Influenza A(H3N2)	4 (2.0)	0.06–3.9
Influenza B	2 (1.0)	0–2.4
Respiratory syncytial virus	23 (11.4)	7.7–16.6
Rhinovirus	36 (17.9)	13.1–23.8
Adenovirus	29 (14.4)	10.2–20.0
Human parainfluenza virus	9 (4.5)	1.6–7.3
Human parainfluenza virus ‐1	2 (1.0)	0.2–3.9
Human parainfluenza virus ‐3	3 (1.5)	0.4–4.5
Human parainfluenza virus ‐4	4 (2.0)	0.7–5.2
Human metapneumovirus	15 (7.4)	4.5–12.0
Enterovirus	1 (0.5)	0.0–3.4
Bocavirus	7 (3.4)	1.6–7.1
Human coronavirus	14 (7.4)	4.1–11.7
Coronavirus NL63	2 (1.0)	0.0–3.9
Coronavirus HKU1	10 (5.0)	2.6‐–9.0
Coronavirus OC43	2 (1.0)	0.0–3.9
Number of co‐infections		
None (SARS‐CoV‐2 only)	98 (48.8)	41.8–55.6
One pathogen	68 (33.8)	27.5–40.6
Two pathogens	24 (11.9)	8.1–17.2
Three pathogens	6 (3.0)	0.3–6.5
Four pathogens	5 (2.4)	0.1–5.8

Abbreviation: CI = confidence interval.

Logistic regression analysis identified several factors independently associated with the presence of viral co‐infection (Table 3). Children aged 6 months to 14 years had a nearly three‐fold higher risk of co‐infection compared to infants under 6 months (aOR = 2.7; 95% CI: 1.2–5.9; *p* = 0.013). Clinical Presentation: The presence of fever ≥ 38°C at admission was strongly associated with the detection of co‐pathogens (aOR = 2.6; 95% CI: 1.2–5.4; *p* = 0.008). Severity: Hospitalization status was the strongest predictor, with hospitalized patients being four times more likely to harbor a co‐infection (aOR = 4.3; 95% CI: 1.5–12.0; *p* = 0.005). In contrast, sex, area of residence, and seasonality did not show a statistically significant association with co‐infection risk after adjustment for confounders.

**Table 3:** Factors associated with viral co‐infections in SARS‐CoV‐2 patients.

Variables	SARS‐CoV‐2 with co‐infection (*n* = 98)	Crude OR (95% CI)	*p*‐value	Adjusted OR (95% CI)	*p*‐value
Age group					
0–6 Months	21 (46.7)	1 (Reference)	—	1 (Reference)	—
6 Months–14 Years	63 (58.9)	1.639 (0.811–3.298)	**0.168**	2.706 (1.229–5.958)	**0.013**
≥ 15 Years	17 (37)	0.669 (0.289–1.547)	0.349	1.252 (0.472–3.320)	0.650
Sex					
Male	42 (49.4)	1 (Reference)	—		
Female	61 (52.6)	1.135 (0.648–1.987)	0.657		
Area of residence					
Rural	9 (63.3)	1 (Reference)	—		
Urban	94 (50)	0.444 (0.132–1.493)	**0.190**		
Region					
Guiriko	77 (56.6)	1 (Reference)	—	1 (Reference)	—
Kadiogo	22 (36.6)	0.443 (0.237–0.828)	**0.011**	0.513 (0.249–1.058)	0.071
Oubri	4 (80)	3.064 (0.333–28.146)	0.322	0.919 (0.070–11.941)	0.949
Vaccination anti‐COVID‐19					
No	5 (62.5)	1 (Reference)	—		
Yes	3 (37.5)	0.558 (0.129–2.400)	0.433		
Malnutrition					
No	8 (61.54)	1 (Reference)	—		
Yes	5 (38.46)	0.571 (0.180–1.814)	0.338		
Fever					
History of fever	61 (43.8)	1 (Reference)	**—**	1 (Reference)	**—**
Fever (≥ 38°C)	78 (56.1)	1.892 (1.030–3.476)	**0.040**	2.653 (1.286–5.471)	**0.008**
Lethargy					
No	1 (33.3)	1 (Reference)	—		
Yes	2 (66.6)	1.920 (0.171–21.527)	0.597		
Cough					
No	95 (48.2)	1 (Reference)	—		
Yes	102 (51.7)	3.221 (0.329–31.502)	0.315		
Dyspnea					
No	2 (66.6)	1 (Reference)	—		
Yes	1 (33.3)	0.470 (0.401–5.274)	0.541		
Respiratory distress					
No	9 (37.5)	1 (Reference)	—		
Yes	15 (62.5)	1.704 (0.708–4.100)	0.233		
Sore throat					
No	6 (75)	1 (Reference)	—		
Yes	2 (25)	0.306 (0.063–1.557)	**0.154**		
Convulsion					
No	2 (50)	1 (Reference)	—		
Yes	2 (50)	0.950 (0.131–6.882)	0.960		
Diarrhea					
No	10 (58.8)	1 (Reference)	—		
Yes	7 (41.1)	0.641 (0.234–1.758)	0.388		
Vomiting					
No	7 (46.6)	1 (Reference)	—		
Yes	8 (53.3)	1.094 (0.381–3.141)	0.866		
Loss of taste					
No	3 (75)	1 (Reference)	—		
Yes	1 (25)	1.09 (0.38–3.14)	0.866		
Loss of smell					
No	3 (100)	1 (Reference)	—		
Yes	0 (0)	0.310 (0.031–3.036)	0.315		
Hospitalization					
No	17 (37.7)	1 (Reference)	—	1 (Reference)	
Yes	28 (62.2)	1.778 (0.901–3.509)	**0.097**	4.311 (1.545–12.032)	**0.005**
Antibiotic before admission					
No	21 (42.8)	1 (Reference)	—		
Yes	28 (57.1)	1.368 (0.715–2.619)	0.343		
Antibiotic during hospitalization					
No	19 (41.3)	1 (Reference)			
Yes	27 (58.7)				
Abnormal chest X‐ray					
No	7 (87.5)	1 (Reference)	**—**		
Yes	1 (12.5)	0.127 (0.015–1.055)	**0.056**		
Oxygen therapy					
No	6 (54.5)	1 (Reference)	—		
Yes	5 (45.4)	0.782 (0.230–2.651)	0.693		
Admitted to ICU					
No	6 (50)	1 (Reference)	‐‐		
Yes	6 (50)	0.948 (0.295–3.046)	0.929		
Season^a^					
Dry season	27 (47.3)	1 (Reference)	—		
Rainy season	30 (52.6)	1.080 (0.584–1.996)	0.804		

*Note:* Bold values indicate statistically significant *p* < 0.05.

Abbreviations: aOR = ajusted odds ratio, CI = confidence intervals, ICU = intensive care unit, OR = odds ratio.

^a^ Dry season: October‐April; Rainy season: May‐September.

## 4 Discussion

This study, involving 201 SARS‐CoV‐2‐positive patients in Burkina Faso, represents the first nationwide sentinel surveillance analysis of respiratory viral co‐infections in a low‐resource setting with limited diagnostic infrastructure. Our findings revealed that 33.8% of COVID‐19 cases were co‐infected with at least one additional respiratory virus, highlighting the common burden and potential clinical implications of viral co‐circulation in the region [14]. This rate exceeds the global average reported in several studies, which ranges from less than 5% to over 29.4% [15, 16, 17], although it remains within the range of 47.2%–57.3% reported in specific high‐prevalence or multiplex testing settings [18, 19]. Such discrepancies likely stem from variations in geographical location, seasonal viral circulation, and the high sensitivity of the multiplex PCR assays employed [20]. These findings contribute to the limited body of evidence from sub‐Saharan Africa, where multiplex respiratory virus surveillance remains scarce.

The most frequently detected co‐pathogens were Rhinovirus (17.9%), Adenovirus (14.4%), and RSV (11.4%), a profile consistent with year‐round viral circulation in tropical regions [21, 22]. Influenza viruses were identified in 10.0% of cases, predominantly Influenza A (9.0%), reflecting established regional seasonal patterns  [23]. Other notable co‐detections included HMPV (7.5%), Bocavirus (3.5%), and seasonal coronaviruses HKU1 (5.0%). This diverse viral landscape underscores the endemic nature of multiple respiratory pathogens in West Africa, where transmission dynamics are heavily influenced by climatic factors such as humidity and temperature [24, 25]. Viral co‐infections may involve competitive or synergistic interactions, potentially influencing viral replication dynamics and host immune responses.

Multivariate analysis identified age, fever, and hospitalization as independent predictors of viral co‐infection. Children aged 6 months to 14 years exhibited significantly higher odds of co‐infection (aOR: 2.7; 95% CI: 1.2–6.0; *p* = 0.013), consistent with their role as primary reservoirs of respiratory viruses due to frequent social exposure and relative immunological immaturity [26, 27, 28, 29]. Fever (≥ 38°C) was strongly associated with co‐infection (aOR: 2.7; 95% CI: 1.3–5.5; *p* = 0.008), suggesting a higher systemic inflammatory response or cumulative viral burden in poly‐infected individuals [30, 31, 32].

Hospitalization emerged as the strongest predictor of co‐infection (aOR: 4.3; 95% CI: 1.5–12.0; *p* = 0.005). However, because hospitalization may represent a marker of disease severity rather than a true exposure variable, the directionality of this association should be interpreted cautiously. Temporal relationships cannot be definitively established due to the cross‐sectional design; it remains unclear whether co‐infection contributes to increased severity or whether more severe cases are simply more likely to be hospitalized and detected.

Conversely, variables such as sex, nutritional status, and vaccination status did not reach statistical significance. While malnutrition is a documented risk factor elsewhere [26, 33, 34, 35], its lack of association here may be attributed to our specific sample distribution. Furthermore, no significant seasonal effect was observed, despite 71.6% of cases occurring during the dry season, a period often associated with increased indoor crowding and lower humidity [36, 37].

Despite the viral etiology, 30.8% of patients received antibiotics during hospitalization, predominantly ceftriaxone (93.6%). This highlights the critical need for antimicrobial stewardship in settings with limited diagnostics. Expanded access to rapid multiplex molecular testing could significantly reduce unnecessary antibiotic prescriptions and support rational clinical decision‐making [38, 39, 40].

Strengths of this study include the use of a structured national sentinel network, standardized case definitions, and comprehensive multiplex diagnostics in a real‐world low‐resource context. Limitations include the relatively small number of SARS‐CoV‐2‐positive cases; however, this sample reflects all laboratory‐confirmed cases detected within the national sentinel surveillance system during 2023, ensuring representativeness at the national level. The modest sample size may nevertheless limit statistical power for rare variables. The cross‐sectional design precludes causal inference. Additionally, the predominantly pediatric composition of the cohort limits extrapolation of these findings to adult populations.

A critical consideration in interpreting these findings is the potential for prolonged viral shedding [41, 42]. Since SARS‐CoV‐2 RNA can remain detectable by RT‐PCR for several weeks following the initial infection, it is possible that in some co‐infected cases, the acute clinical presentation particularly in children was primarily driven by the co‐pathogen (such as RSV or HRV) [43, 44], while the SARS‐CoV‐2 detection represented residual shedding rather than active disease. Conversely, the presence of multiple viruses may also lead to viral interference or synergistic effects that modulate the systemic inflammatory response [45, 46]. The absence of viral load quantification and systematic bacterial testing further limits the ability to clarify temporal relationships and fully characterize the infection landscape.

These findings emphasize the importance of integrated, multiplex‐based respiratory surveillance platforms in low‐resource settings, particularly for pediatric and hospitalized populations. Future research should adopt longitudinal designs, incorporate viral load quantification and bacterial diagnostics, and evaluate the clinical impact of multi‐pathogen infections to inform evidence‐based strategies for pediatric care and resource allocation in Burkina Faso and similar contexts.

## 5 Conclusion

Respiratory viral co‐infections are common among SARS‐CoV‐2 patients in Burkina Faso, particularly in children. The independent associations with fever and hospitalization suggest a link with markers of clinical severity. These findings support integration of multiplex respiratory virus testing into routine surveillance to better inform antimicrobial stewardship and outbreak preparedness.

## Author Contributions

**Benjamin W. O. Kaboré:** investigation, writing – original draft, methodology, writing – review and editing, data curation. **Nina Gouba:** conceptualization, writing – review and editing, supervision, methodology. **Abdoul Kader Ilboudo:** writing – review and editing, data curation, formal analysis. **Assana Cissé:** investigation, writing – review and editing. **Moussa Lingani:** writing – review and editing. **Cathérine Sawadogo:** investigation, writing – review and editing. **Ezéchiel Wendinda Ouédraogo:** writing – review and editing. **Jean Bienvenue Ouoba:** writing – review and editing. **Madi Savadogo:** writing – review and editing. **Zékiba Tarnagda:** funding acquisition, resources, writing – review and editing.

## Funding

The authors received no specific funding for this work.

## Disclosure

The lead author Benjamin W. O. Kaboré affirms that this manuscript is an honest, accurate, and transparent account of the study being reported; that no important aspects of the study have been omitted; and that any discrepancies from the study as planned (and, if relevant, registered) have been explained.

## Conflicts of Interest

The authors declare no conflicts of interest.

## Supporting information

Supporting File 1

Supporting File 2

## Data Availability

The datasets used and/or analyzed during the current study are available from the corresponding author on reasonable request.
