# Correction to “Respiratory Viral Co‐Infections in SARS‐CoV‐2 Positive Patients in Burkina Faso: A Cross‐Sectional Study”

**DOI:** 10.1002/hsr2.73074

**Published:** 2026-08-18

Kaboré BWO, Gouba N, Ilboudo AK, Cissé A, Lingani M, Sawadogo C, Ouédraogo EW, Ouoba JB, et al. Respiratory Viral Co‐Infections in SARS‐CoV‐2 Positive Patients in Burkina Faso: A Cross‐Sectional Study. Health Science Reports. 2026; DOI:10.1002/hsr2.72339.

In the Abstract and Results, the prevalence of viral co‐infections was incorrectly reported as 48.8% in the Abstract and Results sections. This value corresponds to SARS‐CoV‐2 mono‐infected patients rather than co‐infected cases.

Accordingly, in the Results section of the Abstract, the sentence “Co‐infections was identified in 48.8% (95% CI: 41.8–55.6) of cases and a single co‐infections was detected in 33.8% (95% CI: 27.5–40.6) of patients.” was incorrect. It should read: “Viral co‐infections were identified in 51.2% (95% CI: 44.4–58.0) of cases and a single co‐infection was detected in 33.8% (95% CI: 27.5–40.6) of patients.”

In the second paragraph in the Results, the sentence “Overall prevalence of viral co‐infections was 48.8% and 33.8% of patients had a single additional pathogen detected, while 17.4% exhibited multiple co‐infections (ranging from two to four additional viruses) (Table 2).” was incorrect. It should read: “Overall prevalence of viral co‐infections was 51.2% and 33.8% of patients had a single additional pathogen detected, while 17.4% exhibited multiple co‐infections (ranging from two to four additional viruses) (Table 2).”

This correction does not affect the statistical analyses, interpretation of results, or overall conclusions of the study.

We apologize for this error.

