# Supplementary material for: Respiratory Viral Co‐Infections in SARS‐CoV‐2 Positive Patients in Burkina Faso: A Cross‐Sectional Study
Source: Health Sci Rep. 2026 Apr 16;9(4):e72339. doi: 10.1002/hsr2.72339 (PMC13086627; doi:10.1002/hsr2.72339)
Supplement: Supplementary file 2 — Supporting File 2 [file HSR2-9-e72339-s002.pdf]

# Supplementary File 2: Standardized case report forms

**INDIVIDUAL NOTIFICATION FORM FOR SG/SARI/COVID-19 CASES** (Ministry of Health - Burkina Faso)
**Health Facility:** ___________ **District:** ___________ **Region:** ___________
**Care Unit/Service:** ☐ Medicine ☐ Pediatrics ☐ Maternity ☐ Emergency ☐ Other (specify) ___________
**Category:** ☐ ILI (Influenza-Like Illness) ☐ SARI (Severe Acute Respiratory Infection) ☐ COVID-19

**UNIQUE IDENTIFIER (EPID): BFA / __ / __ / __ / __ / SG / SARI / COVID-19 - __**
*(To be completed at the District CISSE level: Country / Region / District / Year / Disease / Case No.)*
**Sticker No.:** ___________ **Epidemiological Week:** ___________☐ Routine Surveillance ☐ Active Case Finding

**PATIENT IDENTIFICATION**
**Last Name:** ___________ **First Name(s):** ___________
**Profession:** ___________ **Date of Birth:** __/__/____
**Age in years:** ___ **or Age in months (if < 12 mo):** ___ **or Age in days (if < 1 mo):** ___
**Sex:** ☐ Male ☐ Female
**Travel history in the 14 days preceding the onset of illness:** ☐ Yes ☐ No
**District of residence:** ___________ **City/Village:** ___________ **Neighborhood/Sector:** ___________ ☐ Urban / ☐ Rural
**Name of father/mother or guardian (if minor):** ___________
**Phone number of patient or parents/guardians:** ___________

**Date of illness onset:** __/__/____ **Date of consultation:** __/__/____**Hospitalized:** ☐ YES ☐ NO **IF YES, Date of hospitalization:** __/__/____
**Antibiotic treatment before hospitalization:** ☐ YES ☐ NO
**SYMPTOMS**
☐ Current Fever OR ☐ History of Fever | **Current Temperature:** ___°C | ☐ Cough
☐ Sore throat ☐ Respiratory difficulties ☐ Diarrhea ☐ Vomiting ☐ Lethargy or unconsciousness
☐ Convulsion ☐ Loss of taste ☐ Inability to drink or eat ☐ Loss of smell
☐ Breathlessness, shortness of breath ☐ Oxygen saturation: ___% ☐ Another sign (specify) ___________
**MEDICAL HISTORY / RISK FACTORS**
☐ Asthma ☐ Tuberculosis ☐ Sickle cell disease ☐ HIV ☐ Diabetes ☐ Obesity ☐ Hypertension ☐ Current pregnancy
☐ SAM + complications (Severe Acute Malnutrition) ☐ MAM (Moderate Acute Malnutrition) ☐ Smoker (Number of packs/year): ___
☐ Other chronic diseases (specify): ___________
**ADDITIONAL EXAMINATIONS**
**Chest X-ray:** ☐ YES ☐ NO | **IF YES, results:** ☐ Normal ☐ Infiltrates ☐ Opacities ☐ Cavities
☐ Pleurisy ☐ Others (specify): ___________
**Malaria RDT/Blood Smear:** Result ☐ Positive ☐ Negative
**TREATMENT AND EVOLUTION**
**Antibiotic treatment during hospitalization:** ☐ YES ☐ NO. *IF YES: specify the antibiotic(s) administered:* ___________
☐ Oxygen therapy ☐ Other treatments (specify): ___________
**Evolution:** ☐ Recovered ☐ Deceased ☐ In treatment ☐ Unknown | **Date of outcome:** __/__/____
☐ Evacuated; If evacuated, to which hospital: ___________ | **Date of evacuation:** __/__/____
*If Evolution Unknown, specify type of discharge:* ☐ Discharge without medical advice ☐ Discharge against medical advice

**ORAL CONSENT:** ☐ YES ☐ NO *I acknowledge that I have been duly informed of the sampling and treatment procedures on myself or my child. I signify my agreement for the sampling and necessary tests.*

**VACCINATION STATUS**
**Flu vaccine:** ☐ YES ☐ NO | **Date of vaccination:** __/__/____
**Anti-COVID-19:** ☐ YES ☐ NO | **Date of vaccination:** __/__/____
**DTC-HepB-Hib1 (penta1):** ☐ YES ☐ NO [Date __/__/____]
**Pneumo1 (PCV13):** ☐ YES ☐ NO [Date __/__/____]
**DTC-HepB-Hib2 (penta2):** ☐ YES ☐ NO [Date __/__/____]
**DTC-HepB-Hib3 (penta3):** ☐ YES ☐ NO [Date __/__/____]
**Pneumo2 (PCV13):** ☐ YES ☐ NO [Date __/__/____]
**Pneumo3 (PCV13):** ☐ YES ☐ NO [Date __/__/____]
**Source of info:** ☐ Oral ☐ Vaccination card/booklet or other written proof

**SAMPLE COLLECTED**
☐ YES ☐ NO *(Note: IF NO, still complete the form and forward it to the district CISSE)*
**IF YES: Date of collection:** __/__/____ | **Time of collection:** __H__Min
**Nature of sample:** ☐ Oropharyngeal ☐ Nasopharyngeal
**Sample preservation before transport:** ☐ Ambient temperature ☐ Fresh (+2°C to +8°C) ☐ Frozen
**Date of sample receipt at district lab:** __/__/____ | **Time:** __H__Min
**La Poste Burkina Faso Parcel No.:** ___________
**Date sent to the National Reference Laboratory:** __/__/____ | **Time of dispatch:** __H__Min
**IF NO: Why?** ☐ Lack of kit ☐ Lack of competence ☐ Patient condition ☐ Patient refusal ☐ Others (specify): ___________

**Name of agent who completed the form:** ___________ **Tel:** ___________
**Date form was completed:** __/__/____
**Date form sent to District:** __/__/____ **Date form received at District:** __/__/____

**LABORATORY RESULTS**
**NATIONAL REFERENCE LABORATORY FOR INFLUENZA**
**Date of receipt:** __/__/____ **Time:** __H__Min
**La Poste Burkina Faso Parcel No.:** ___________ **Lab Register No.:** ___________ **Sticker No.:** ___________
**Temperature at receipt:** ☐ Frozen ☐ Fresh (+2°C to +8°C) ☐ Ambient temperature
**Condition of sample at receipt:** ☐ Adequate ☐ Inadequate
**PCR performed:** ☐ YES ☐ NO | **IF YES:**
**Date of Influenza PCR:** __/__/____
**Influenza PCR results:** ☐ Positive ☐ Negative
**Sub-typing Date:** __/__/____ | ☐ Influenza A ☐ A(H1N1) pdm09 ☐ A(H1N1) ☐ A(H3N2) ☐ Influenza B ☐ B(Victoria) ☐ B(Yamagata)
☐ Non-subtypable ☐ Non-subtyped ☐ Other sub-type (specify): ___________
**Date of RSV PCR:** __/__/____ | **Results:** ☐ RSV | **Sub-type:** ☐ RSV A ☐ RSV B
**Date of COVID-19 PCR:** __/__/____ | **COVID-19 PCR results:** ☐ Positive ☐ Negative
**SARS-CoV-2 Screening Date:** __/__/____ | **Result (variant):** ___________
**Sequencing:** ☐ YES ☐ NO | **IF YES: Sequencing Date:** __/__/____ | **Result (variant):** ___________

**MULTIPLEX PCR RESULTS - OTHER RESPIRATORY PATHOGENS**

| Panel | Pathogens | Results |
|---|---|---|
| N° 1 : | Influenza A (FluA) | ☐ Positive ☐ Negative ☐ Indeterminate |
| | Influenza B (FluB) | ☐ Positive ☐ Negative ☐ Indeterminate |
| | Influenza A (H1N1) swl | ☐ Positive ☐ Negative ☐ Indeterminate |
| | Rhinovirus | ☐ Positive ☐ Negative ☐ Indeterminate |
| N° 2 : | Coronavirus NL63 | ☐ Positive ☐ Negative ☐ Indeterminate |
| | Coronavirus 229E | ☐ Positive ☐ Negative ☐ Indeterminate |
| | Coronavirus OC43 | ☐ Positive ☐ Negative ☐ Indeterminate |
| | Coronavirus HKU1 | ☐ Positive ☐ Negative ☐ Indeterminate |
| N° 3 : | Parainfluenza 2 | ☐ Positive ☐ Negative ☐ Indeterminate |
| | Parainfluenza 3 | ☐ Positive ☐ Negative ☐ Indeterminate |
| | Parainfluenza 4 | ☐ Positive ☐ Negative ☐ Indeterminate |
| N° 4 : | Parainfluenza 1 | ☐ Positive ☐ Negative ☐ Indeterminate |
| | Human metapneumovirus A/B | ☐ Positive ☐ Negative ☐ Indeterminate |
| | Bocavirus | ☐ Positive ☐ Negative ☐ Indeterminate |
| | *Mycoplasma pneumoniae* | ☐ Positive ☐ Negative ☐ Indeterminate |
| N° 5 : | Respiratory syncytial viruses A/B | ☐ Positive ☐ Negative ☐ Indeterminate |
| | Adenovirus | ☐ Positive ☐ Negative ☐ Indeterminate |
| | Enterovirus | ☐ Positive ☐ Negative ☐ Indeterminate |
| | Parechovirus | ☐ Positive ☐ Negative ☐ Indeterminate |
| N° 6 : | *Chlamydia pneumoniae* | ☐ Positive ☐ Negative ☐ Indeterminate |
| | *Streptococcus pneumoniae* | ☐ Positive ☐ Negative ☐ Indeterminate |
| | *Haemophilus influenzae* type B | ☐ Positive ☐ Negative ☐ Indeterminate |
| | *Staphylococcus aureus* | ☐ Positive ☐ Negative ☐ Indeterminate |
| N° 7 : | *Klebsiella pneumoniae* | ☐ Positive ☐ Negative ☐ Indeterminate |
| | *Legionella pneumophila/Legionella longbeachae* | ☐ Positive ☐ Negative ☐ Indeterminate |
| | *Salmonella spp* | ☐ Positive ☐ Negative ☐ Indeterminate |
| | *Pneumocystis jirovecii* | ☐ Positive ☐ Negative ☐ Indeterminate |
| N° 8 : | *Moraxella catarrhalis* | ☐ Positive ☐ Negative ☐ Indeterminate |
| | *Bordetella spp.* (except *Bordetella parapertussis*) | ☐ Positive ☐ Negative ☐ Indeterminate |
| | *Haemophilus influenzae* | ☐ Positive ☐ Negative ☐ Indeterminate |
| | Influenza C (FluC) | ☐ Positive ☐ Negative ☐ Indeterminate |

**Other test (specify type and results):** ___________
**Observations:** ___________
**Date of transmission of results to DPSP (Direction de la protection de la santé des populations) :** __/__/____
